# Disease Resistance Gene Analogs (RGAs) in Plants

**DOI:** 10.3390/ijms160819248

**Published:** 2015-08-14

**Authors:** Manoj Kumar Sekhwal, Pingchuan Li, Irene Lam, Xiue Wang, Sylvie Cloutier, Frank M. You

**Affiliations:** 1 Cereal Research Centre, Agriculture and Agri-Food Canada, Morden, MB R6M 1Y5, Canada; E-Mails: manoj.sekhwal@agr.gc.ca (M.K.S.); pingchuan.li@agr.gc.ca (P.L.); irene.lam@agr.gc.ca (I.L.); 2 National Key Laboratory of Crop Genetics and Germplasm Enhancement, Cytogenetics Institute, Nanjing Agricultural University, Nanjing 210095, China; E-Mail: xiuew@njau.edu.cn; 3 Eastern Cereal and Oilseed Research Centre, Agriculture and Agri-Food Canada, Ottawa, ON K1A 0C6, Canada; E-Mail: sylvie.j.cloutier@agr.gc.ca; 4 Plant Science Department, University of Manitoba, Winnipeg, MB R3T 2N6, Canada

**Keywords:** disease resistance gene, gene mining, nucleotide binding site leucine rich repeat (NBS-LRR), pentatricopeptide repeats (PPRs), resistance gene analog (RGA), receptor like kinase (RLK), receptor like protein (RLP), small RNA (sRNA)

## Abstract

Plants have developed effective mechanisms to recognize and respond to infections caused by pathogens. Plant resistance gene analogs (RGAs), as resistance (*R*) gene candidates, have conserved domains and motifs that play specific roles in pathogens’ resistance. Well-known RGAs are nucleotide binding site leucine rich repeats, receptor like kinases, and receptor like proteins. Others include pentatricopeptide repeats and apoplastic peroxidases. RGAs can be detected using bioinformatics tools based on their conserved structural features. Thousands of RGAs have been identified from sequenced plant genomes. High-density genome-wide RGA genetic maps are useful for designing diagnostic markers and identifying quantitative trait loci (QTL) or markers associated with plant disease resistance. This review focuses on recent advances in structures and mechanisms of RGAs, and their identification from sequenced genomes using bioinformatics tools. Applications in enhancing fine mapping and cloning of plant disease resistance genes are also discussed.

## 1. Introduction

Many plant-pathogen interactions are determined by the presence of resistance (*R*) genes/alleles that enable plants to recognize pathogens and activate inducible defenses [1]. Plant diseases have been reported to be caused by a wide variety of pathogens, such as *Fusarium* sp. in fusarium head blight (FHB) [2], *Sitodiplosis mosellana* in wheat midge [3], *Blumeria* sp., *Erysiphe Golovinomyces* sp. and *Oidium* in powdery mildew [4], *Puccinia* sp. in rust [5], *Phytophthora infestans* in late blight [6], and *Pseudomonas syringae* in bacterial speck [7]. Plants have developed effective mechanisms to recognize and respond to infections with race non-specific resistance (quantitative) and race-specific resistance (qualitative). Race non-specific resistance involves a number of minor genes that are effective against several pathogens [8]. For example, *NDR1* and three alleles of *rp1*, *rp1-D21*, *rp1-MD19* and *rp-NC3*, have been shown to confer a race non-specific resistance response to rust [9]. In contrast, race-specific resistance is conferred by a single or a few major genes that operate in a gene-for-gene fashion in which plant *R-*genes recognize corresponding pathogen avirulence (*Avr)*-gene effectors to trigger plant defense responses [10]. *Pto* in tomato confers race-specific resistance to *Pseudomonas syringae* pv. tomato (Pst), similarly to *RPS2* in *Arabidopsis* and *N* (mosaic virus resistance gene) in tobacco [11]. On the other hand, the wheat stem rust resistance gene *Sr26* shows resistance to all races of the pathogen *Puccinia graminis* f. sp. *tritici*, thus constituting a race non-specific pathosystem [12].

In the defense system of plants, direct and indirect interactions are two alternative mechanisms to explain the gene-for-gene model. In the direct interaction, pathogen Avr effectors associate directly with plant *R*-genes to trigger signaling. For example, rice *R*-gene *Pi-ta* was shown to directly interact with *Avr-Pita* from *Magnaporthe grisea* [13]. Likewise, a direct interaction was observed between *L* genes (a group of resistant genes to flax rust) and their corresponding rust *Avr* genes in flax [14]. The indirect model is also known as the guard hypothesis [10]. In this model, *R*-gene products act as a “guardee” to monitor the modification of host proteins after associating with the pathogenic effectors, resulting in the initiation of resistance [15]. Examples of guardee proteins are RPM1 interacting 4 (RIN4), flagellin-sensitive 2 (FLS2), Xa21, resistance to pseudomonas syringae 5 (RPS5) and avrPphB susceptible 1 (PBS1). Resistance gene analogs (RGAs) are a large class of potential *R***-**genes that have conserved domains and structural features. As such, RGAs can be identified from sequenced genomes using bioinformatics approaches [16,17,18]. In the last 15 years, more than 50 plant genomes have been sequenced and assembled [19,20,21]. Despite this great resource, only a relatively small number of *R*-genes have been cloned and fully characterized (Table 1) providing not only information on their structure, function and evolution, but also generating useful genetic resources to create novel resistant cultivars [22]. In comparison, thousands of RGAs have been identified in many plant genomes (Table 2).

**Table 1:** Cloned *R-*genes from plant species.

Species ^a^	*R*-Gene	Accession ID	Domain ^b^	Class	Chr	Disease ^c^	*Avr* Gene	Pathogen ^c^	Reference
*T. aestivum* (wheat)	*Lr10*	AAQ01784	CNL	NBS	1A	Leaf rust	*AvrLr10*	*P. triticina*	[23]
	*Lr1*	ABS29034	CNL	NBS	5D	Leaf rust	*Avr1*	*P. triticina*	[24]
	*Pm3b*	AAQ96158	CNL	NBS	1A	Powdery mildew	*AvrPm3b*	*B. graminis*	[25]
	*Sr33*	AGQ17390	CNL	NBS	1D	Stem rust	**	*P. graminis*	[26]
	*Sr35*	AGP75918	CNL	NBS	3A	Stem rust	**	*P. graminis*	[27]
	*Lr21*	AAP74647	NL	NBS	1D	Leaf rust	*AvrLr21*	*P. triticina*	[28]
	*Cre3*	AAC05834	NL	NBS	2D	Cereal cyst	**	*H. avenae*	[29]
	*Cre1*	AAM94164	NL	NBS	2B	Cereal cyst	**	*H. avenae*	[30]
	*Yr10*	AAG42168	CNL	NBS	1B	Stripe rust	**	*P. striiformis*	[31]
	*Stpk-V (Pm21)*	AEF30547	STK	Oth-R	6V	Powdery mildew	**	*B. graminis*	[32]
	*Lr34*	ACN41354	*ABC transporter*	Oth-R	7D	Leaf rust, Powdery mildew, Stripe rust	**	*P. triticina*; *P. striiformis*; *B. graminis*	[33]
	*Yr36*	ACF33195	Kinase-START	Oth-R	6B	Stripe rust	**	*P. striiformis*	[34]
*H. vulgare* (barley)	*Mla6*	CAC29241	CNL	NBS	1	Powdery mildew	*AvrMla6*	*B. graminis*	[35]
	*Mla1*	AAG37356	CNL	NBS	1	Powdery mildew	*AvrMla1*	*B. graminis*	[36]
	*Mla13*	AAO16014	CNL	NBS	1	Powdery mildew	*AvrMla13*	*B. graminis*	[37]
	*Rpg1*	AAM81980	LRR-PK	RLK	7	Stem rust	*Avr-Rpg1*	*P. graminis*	[38]
	*Mlo*	CAB06083	TM	Oth-R	4	Powdery mildew	**	*E. graminis*	[39]
*S. lycopersicum* (tomato)	*Prf*	AAC49408	CNL	NBS	5	Bacterial speck	*AvrPto*	*P. syringae*	[40]
	*Mi*	AAC67238	CNL	NBS	6	Root knot	**	*M. javanica*	[41]
	*I2*	AAB63274	NL	NBS	11	Fusarium wilt	*Avr1*	*F. oxysporum*	[42]
	*Ph*-*3*	AIB02970	CNL	NBS	9	Late blight	**	*P. infestans*	[43]
	*Sw-5*	AAG31013	CNL	NBS	9	Tomato spotted wilt	**	TSWV	[44]
	*Tm-2*	AAQ10735	CNL	NBS	9	Tobacco mosaic		TMV	[45]
	*Bs4*	AAR21295	TNL	NBS	5	Bacterial spot	*AvrBs4*	*X. campestris*	[46]
	*Hero*	CAD29729	CNL	NBS	4	Potato cyst	**	*G. rostochiensis*	[47]
	*Cf-2*	2207203A	LRR-TM	RLP	6	Leaf mold	*Avr2*	*C. fulvum*	[48]
	*Cf-4*	CAA05268	LRR-TM	RLP	1	Leaf mold	*Avr4*	*C. fulvum*	[49]
	*Cf-5*	AAC78591	LRR-TM	RLP	6	Leaf mold	*Avr5*	*C. fulvum*	[50]
	*Cf-9*	CAA05274	LRR-TM	RLP	1	Leaf mold	*Avr9*	*C. fulvum*	[51]
	*Ve1*,*2*	AAK58681.2	LRR-TM	RLP	9	Verticillium wilt	**	*V. dahliae*	[52]
	*Hcr9-4E*	CAA05269	LRR-TM	RLP	1	Leaf mold	*Avr4E*	*C. fulvum*	[49]
	*Fen*	2115395A	STK	Oth-R	5	Bacterial speck	*AvrPto*	*P. syringae*	[53]
	*Pto*	A49332	STK	Oth-R	5	Bacterial speck	*AvrPto*	*P. syringae*	[54]
	*Pti1*	NP_001233803	STK	Oth-R	12	Bacterial speck	**	*P. syringae*	[55]
*S. tuberosum* (potato)	*Rx*	CAB50786	CNL	NBS	12	PVX		PVX	[56]
	*RB*	Q7XBQ9	CNL	NBS	8	Late blight	*Avr1*, *Ipio*, *Ipib*	*P.* *infestans*	[57]
	*Rx2*	CAB56299	LZ-NL	NBS	5	PVX		PVX	[58]
	*R1*	AAL39063	LZ-NL	NBS	5	Late blight	*Avr1*	*P. infestans*	[59]
*L. sativa* (lettuce)	*Rgc2 (Dm3)*	Q9ZSD1	NL	NBS		Downy mildew	*Avr3*	*B. lactucae*	[60]
*P. nigrum* (black pepper)	*Bs2*	AAF09256	CNL	NBS		Bacterial spot	*AvrBs2*	*X. campestris*	[61]
*O. sativa* (rice)	*Xa1*	BAA25068	NL	NBS	4	Bacterial blight	*AvrXoo*	*X. oryzae*	[62]
	*Pib*	BAA76282	CNL	NBS	2	Blast	**	*M. grisea*	[63]
	*Pi-ta*	AAK00132	NL	NBS	12	Blast	*Avr-Pita*	*M. grisea*	[64]
	*Pi36*	ABI64281	CNL	NBS	8	Blast	*Avr-Pi36*	*M. grisea*	[65]
	*Pia*	BAK39926	CNL	NBS	11	Blast	*AvrPia*	*M. oryzae*	[66]
	*Pi-K^h^*	AAY33493	NL	NBS	11	Blast	*Avr-Pik*	*M. oryzae*	[67]
	*Pi37*	ABI94578	NL	NBS	1	Blast	**	*M. grisea*	[68]
	*Xa5*	A2XZI2	NL	NBS	5	Bacterial blight	*AvrXa5*	*X. oryzae*	[69]
	*Xa13*	ABD78944	SET	Oth-R	8	Bacterial blight	*AvrXa13*	*X. oryzae*	[70]
	*Pi54*	CCD33085	CNL	NBS		Blast	*AvrPi54*	*M. oryzae*	[71]
	*Pi9*	ABB88855	CNL	NBS	6	Blast	*AvrPi9*	*M. grisea*	[72]
	*Piz-t/Pi2*	ABC73398	CNL	NBS	6	Blast	*AvrPiz-t*	*M. grisea*	[73]
	*Rpr1*	BAA75812	CNL	NBS	11	Blast	**	*M. grisea*	[74]
	*Pid3*	ACN62386	CNL	NBS	6	Blast	**	*M. grisea*	[75]
	*Xa21*	AAC49123	LRR-STK	RLK	11	Bacterial blight	*AvrXa21*	*X. oryzae*	[76]
	*Xa3/Xa26*	ABD36512	LRR-STK	RLK	11	Bacterial blight	**	*X. oryzae*	[77]
	*CEBiP*	BAE95828		RLK	3		**	**	[78]
	*Xa10*	AGE45112		Oth-R	11	Bacterial blight	*AvrXa10*	*X. oryzae*	[79]
	*Xa25*	AGS56390	TM	Oth-R	12	Bacterial blight	**	*X. oryzae*	[80]
	*Xa27*	AEW90324	LRR-TM	RLP	6	Bacterial blight	*AvrXa27*	*X. oryzae*	[81]
	*Pi-d2*	ACR15163	B-lectin, STK	RLK	6	Blast	**	*M. grisea*	[82]
*Z. mays* (maize)	*Rp1-D*	AAD47197	NL	NBS	10	Rust	**	*P. sorghi*	[83]
	*Hm1*	Q41867			1	Corn leaf blight	**	C. carbonum	[84]
*A. thaliana* (*Arabidopsis*)	*RPM1*	CAA61131	CNL	NBS	3	Downy mildew	*AvrB*, *AvrRpm1*	*P. syringae*	[85]
	*RPS2*	AAA21874	NL	NBS	4	Downy mildew	*AvrRpt2*	*P. syringae*	[86]
	*RPP8/HRT*	AAC83165	CNL	NBS	5	Downy mildew	*AvrRPP8*	*P. parasitica*	[87]
	*RPP13*	AAF42832	CNL	NBS	3	Downy mildew	*ATR13*	*P. parasitica*	[88]
	*RCY1*	BAC67706	CNL	NBS	5	Mosaic type	**	CMV	[89]
	*RPP1*	AAC72977	TNL	NBS	3	Downy mildew	*ATR1*	*P. parasitica*	[90]
	*RPP4*	AAM18462	TNL	NBS	4	Downy mildew	**	*P. parasitica*	[91]
	*RPS4*	CAB50708	TNL	NBS	5	Powdery mildew	*AvrRps4*	*P. syringae*	[92]
	*RPP5*	AAF08790	TNL	NBS	4	Downy mildew	*AvrRp5*	*P. parasitica*	[93]
	*RPS5*	AAC26126	NL	NBS	1	Downy mildew	*AvrRphB*	*P. syringae*	[94]
	*RRS1*	ADM88042	WRKY-TNL	NBS	5	Bacterial wilt	*AvrRRS1*	*R. solanacearum*	[95]
	*RPP27*	CAE51864	LRR-TM	RLP	1	Downy mildew	**	*P. parasitica*	[96]
	*RFO1*	AAY86486	LRR-STK	RLK	1	Fusarium wilt	**	*F. oxysporum*	[97]
	*PBS1*	AAG38109	STK	Oth-R	5		*AvrPphB*	*P. syringae*	[98]
	*FLS2*	AED95370	LRR-STK	RLK	5	Powdery mildew	*AvrPto*, *AvrPtoB*	*P. syringae*	[99]
	*BAK1*	AT4G33430	LRR-STK	RLK	4		*AvrPto*, *AvrPtoB*	*P. syringae*	[100]
	*NDR1*	AAB95208	TM	Oth-R	3		*AvrB*, *AvrRpt2*	*P. syringae*; *P. parasitica*	[101]
	*RPW8*	AAK09267	RPW8	Oth-R	3	Powdery mildew	**	*E. cruciferarum*	[102]
*L. usitatissimum* (flax) ^d^	*L6*	AAA91022	TNL	NBS	5	Rust	*AvrL6*	*M. lini*	[103]
	*L*, *L1-L11*	AAD25974	TNL	NBS	5	Rust	*AvrBs3*	*M. lini*	[104]
	*M*	AAB47618	TNL	NBS	8	Rust	*AvrM*	*M. lini*	[105]
	*P* , *P1-4*	AAK28806	TNL	NBS	14	Rust	**	*M. lini*	[106]
*B. vulgaris* (sugar beet)	*Hs1pro-1*	AAB48305	LRR-TM	RLP	1	Beet cyst	**	*H. schachtii*	[107]
*N. tabacum* (tobacco)	*N*	AAA50763	TNL	NBS		Tobacco mosaic	**	TMV	[108]

^a^: *A. thaliana*, *Arabidopsis thaliana*; *B. vulgaris*, *Beta vulgaris*; *H. vulgare*, *Hordeum vulgare*; *L. sativa*, *Lactuca sativa*; *L. usitatissimum*, *Linum usitatissimum*; *N. tabacum*, *Nicotiana tabacum*; *O. sativa*, *Oryza sativa*; *P. nigrum*, *Piper nigrum*; *S. lycopersicum*, *Solanum lycopersicum*; *S. tuberosum*, *Solanum tuberosum*; *T. aestivum*, *Triticum aestivum*; *Z. mayes*, *Zea mayes*; ^b^: SET, sugar efflux transporter; TM, transmembrane; STK, serine/threonine protein kinase; ^c^: PVX, potato virus X; *B. graminis*, *Blumeria graminis*; *B. lactucae*, *Bremia lactucae*; *C. fulvum*, *Cladosporium fulvum*; *C. carbonum*, *Cochliobolus carbonum*; *E. cruciferarum*, *Erysiphe cruciferarum*; *E. graminis*, *Erysiphe graminis*; *F. oxysporum*, *Fusarium oxysporum*; *G. rostochiensis*, *Globodera rostochiensis*; *H. avenae*, *Heterodera avenae*; *H. schachtii*, *Heterodera schachtii*; *M. grisea*, *Magnaporthe grisea*; *M. oryzae*, *Magnaporthe oryzae*; *M. lini*, *Melampsora lini*; *M. javanica*, *Meloidogyne javanica*; *P. parasitica*, *Peronospora parasitica*; *P. infestans*, *Phytophthora infestans*; *P. syringae*, *Pseudomonas syringae*; *P. graminis*, *Puccinia graminis*; *P. sorghi*, *Puccinia sorghi*; *P. striiformis*, *Puccinia striiformis*; *P. triticina*, *Puccinia triticina*; *R. solanacearum*, *Ralstonia solanacearum*; *V. dahliae*, *Verticillium dahliae*; *X. campestris*, *Xanthomonas campestris*; *X. oryzae*, *Xanthomonas oryzae* pv. *oryzae* (Xoo); CMV, cucumber mosaic virus; TMV, tobacco mosaic virus; TSWV, tomato spotted wilt virus; ^d^: The chromosome numbers of genes were based on unpublished data.

**Table 2:** Genome-wide identification of RGAs in plant genomes.

Species ^a^	Genome Size (Mb) ^b^	Total Annotated Genes ^b^	Total RGAs (%) ^c^	NBS Coding Genes ^d^							PPR ^e^	RLK ^f^	RLP ^g^	Other ^h^	Identification Method Used ^i^	Reference
				CNL	TNL	CN	NL	TN	N	Total						
**Dicots**																
*A. thaliana* (*Arabidopsis*)	125	25,498	5.27	51	79	8	20	17	26	201	441	600	56	46	H, P, B	[109–113]
*A. lyrata* (lyrata)	207	32,670	0.56	21	103	17	14	20	10	185					H, B	[114]
*P. trichocarpa* (black cottonwood)	485	45,555	3.18	119	64	19	83	13	46	344	600	379		127	MEME, CO, Paircoil2, MC	[111,115–117]
*V. vinifera* (grape)	475	30,434	3.81	203	97	26	12	14	0	352	600			210	H, B, MEME	[111,118]
*L. usitatissimum* (flax)	373	43,484	0.34	31	57	10	5	22	7	132				16	MEME/MAST	[119]
*S. lycopersicum* (tomato)	900	34,727	0.84	118	18	19	43	5	49	252		16	13	13	H, B	[120,121]
*C. papaya* (papaya)	372	28,629	0.18	4	6				44	54					TBN, MEME, CW, MC, H	[122]
*C. sativus* (cucumber)	367	26,682	0.26	25	19	1	17	5	3	70					H, CO, ME, CX, SMART, P, B	[123]
*S. tuberosum* (potato)	844	39,031	1.47	65	37	24	184	12	113	435				142	H, B	[124]
*M. truncatula* (*Medicago*)	454	62,388	1.20	152	118	25	0	38	328	661				92	B, H	[111]
*G. raimondii* (cotton)	880	40,976	1.19	35	41	18	96	9	31	230		60	144	56	B, CO, SMART, MC, CW, IPS, ME5,	[125,126]
*B. rapa*, (chinese cabbage)	485	41,174	0.60	19	93	15	27	23	29	206				42	B, H	[111]
*B. oleracea* (cabbage)	630	45,758	0.52	6	40	5	24	29	53	157				82	B, H	[111]
*F. vesca* (strawberry)	240	34,809	0.27		61		16	8	1	86				8	B, MU, ME, MEME	[127]
*M. x domestica* (apple)	742	57,386	1.86	218	161	54	276	69	182	960				110	H, B, CW, MEME	[17]
*L. japonicus* (lotus)	472	19,848	0.42	9	8	19	3	16	29	84					BP, CO, P, MEME	[128]
*T. cacao (*cocoa*)*	430	28,798	1.09	82	8	46	104	4	53	297				17	B, H	[111]
*P. patens* (moss)	510	35,938	0.46	9	3	2	5	0	1	20	103			45	B, CO, MU, ME	[129,130]
Average	500	37,433	1	69	56	19	55	18	56	263	436	264	71	72		
**Monocots**																
*O. sativa* (rice*)*	420	59,855	4.22	159	0	7	40	3	45	254	477	1429	90	281	H, B, MEME, P	[110,130–133]
*T. aestivum* (wheat)	17,000	94,000	2.37	98		0	555		318	971				1266	H, B, MEME	[134]
*Z. mayes* (maize*)*	2300	32,540	0.90	58	0	21	31	0	69	179		113		2	P, H, B, CO	[135,136]
*S. bicolor* (sorghum*)*	739	34,496	1.29	36	0	99	133	0	64	332				114	P, H, B, CO, ME, CW	[137,138]
*H. vulgare* (barley)	5100	30,400	1.38	101		51	145		34	331				89		[139,140]
*B. distachyon* (*Brachypodium*)	272	25,532	1.23	133	0	28	87	0	34	282				34	P, H, B, CO, CW	[140,141]
*T. urartu* (Red wild einkorn)	4940	34,879	1.63	235	0	44	218		38	535				35	H	[140,142]
*A. tauschii* (Tausch’s goatgrass)	4360	43,150	1.94	296	0	63	288		81	728				112	H	[140,143]
Average	4391	44,357	2	140	0	39	187	1	85	452	477	771	90	242		

^a^: *A. tauschii*, *Aegilops tauschii*; *A. lyrata*, *Arabidopsis lyrata*; *B. distachyon*, *Brachypodium distachyon*; *B. oleracea*, *Brassica oleracea*; *B. rapa*, *Brassica rapa*; *C. papaya*, *Carica papaya*; *C. sativus*, *Cucumis sativus*; *F. vesca*, *Fragaria vesca*; *G. raimondii*, *Gossypium raimondii*; *L. japonicus*, *Lotus japonicus*; *M. truncatula*, *Medicago truncatula*; *M. x domestica*, *Malus x domestica*; *P. patens*, *Physcomitrella patens*; *P. trichocarpa*, *Populus trichocarpa*; *S. bicolor*, *Sorghum bicolor*; *T. cacao*, *Theobroma cacao*; *T. urartu*, *Triticum urartu*; *V. vinifera*, *Vitis vinifera*; ^b^: Most of the information concerning the genome sizes and the total number of annotated genes was obtained from [144]; ^c^: The percentages calculated based on present data, not referred from references; ^d^: CNL, CC-NBS-LRR; TNL, TIR-NBS-LRR; CN, CC-NBS; NL, NBS-LRR; TN, TIR-NBS; N, NBS; ^e^: PPR, pentatricopeptide repeat; ^f^: RLK, receptor like kinase; ^g^: RLP, receptors like proteins; ^h^: Other, includes TIRX, XN, TNLX, TNTNL, TTNL, XTNX, CNX, TX and Partial NBS–LRR; ^i^: B, BLAST; CO, COILS; CW, ClustalW; CX, ClustalX; H, HMM; MC, MARCOIL; IPS, InterProScan; ME, MEGA; MU, MUSCLE; P, Pfam.

Though a large number of resistance gene loci have been identified in plants using linkage mapping or association studies, most of them correspond to flanking molecular markers or quantitative trait loci (QTL). Mapped genome-wide RGAs, as *R-*gene candidates, are valuable genomic resources to develop high-density *R-*gene genetic maps, design diagnostic markers and co-localize QTL. The markers designed from RGAs can be used for fine mapping and cloning of *R*-genes and, for breeding purposes. This review focuses on recent advances in studies of the structures and functions of RGAs, their identification using bioinformatics tools and their applications in genetic research and breeding for disease resistance.

## 2. Structure and Functional Mechanisms of Resistance Gene Analogs (RGA)

RGAs can be grouped as either nucleotide binding site leucine rich repeat (NBS-LRR) or transmembrane leucine rich repeat (TM-LRR) [145]. Recent findings have identified other modes of plant resistance mechanisms including pentatricopeptide repeats (PPRs) and peroxidases. NBS-LRR can be further classified as toll/interleukin receptor (TIR)-NBS-LRR (TNL) or non-TNL/coiled coil-NBS-LRR (CNL) [145]. Both TNL and CNL specifically target pathogenic effector proteins inside the host cell, termed effector triggered immunity (ETI) response [146]. Likewise, TM-LRRs can be subdivided into two classes: receptor like kinases (RLKs) and other receptor like proteins (RLPs) [145]. RLPs and RLKs are pattern recognition receptors (PRRs) that mediate pathogen/microbe associated molecular pattern (PAMP/MAMP) triggered immunity (PTI/MTI) to allow recognition of a broad range of pathogens [146]. PAMP/MAMPs are conserved features of most pathogens, such as chitin, flagella, and lipopolysaccharides.

### 2.1. Nucleotide Binding Site Leucine Rich Repeat (NBS-LRR) Family

NBS-LRR is the best-known family of RGAs. The two classes of NBS-LRR are distinguished by their N terminal TIR or non-TIR domains. The non-TIR domains are most commonly coiled coil (CC) structures [147]. Another non-TIR domain is the leucine zipper (LZ), with interspersed hydrophobic heptad repeat sequences L-X(6)-L-X(6)-L-X(6)-L [148]. The domain combination refers to LZ-NBS-LRR proteins [148] which are not as common but have been found in agricultural plants such as tomato and potato [40,59]. At the N-terminal region lies the highly irregular and variable LRR domain [149]. This domain is responsible for protein-protein interactions [150]. Between the NBS and LRR domains exists a region called the ARC domain, named so because of its occurrence in APAF-1, R protein and CED-4 [10]. This ARC domain can be further divided into ARC1 and ARC2 subdomains. The ARC domain, together with the NBS domain, forms a region for nucleotide binding [151].

**Figure 1:**
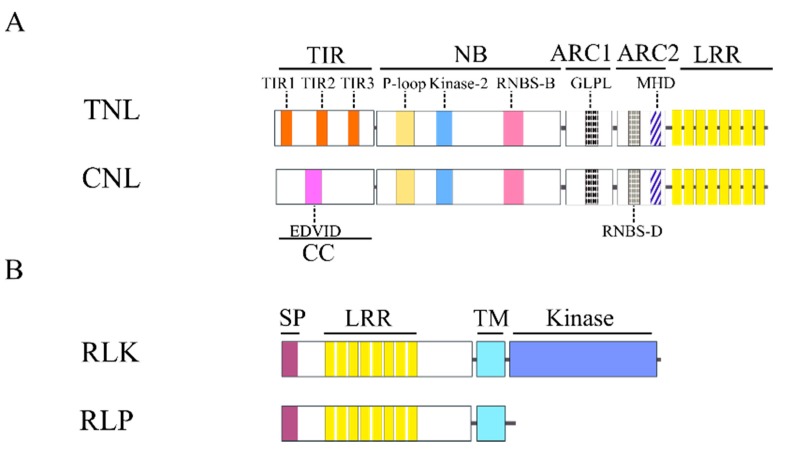
Schematic representation of common structures of four major plant R proteins. Motifs are depicted as colored boxes and labeled under the domain names. Note: the domain lengths are not drawn to scale for ease of visualization. (**A**) Typical domain dissection for TNL and CNL proteins. Only highly conserved motifs are illustrated; (**B**) Domain structures for RLKs and RLPs. The kinase domain is absent in RLPs. Other common domains utilized in our genome-wide identification pipeline are labeled above the colored boxes. TIR: Toll/interleukin-1 receptor; NB: nucleotide-binding site; ARC: abbreviated from Apaf-1, R proteins and CED-4; CC: coiled-coil; SP: signal peptide; TM: transmembrane; LRR: leucine-rich repeats.

Various conserved motifs exist within domains and subdomains of TNL and CNL [152]. The pentapeptide EDVID (EDxxD) motif, denoted as CC_D_, can be identified in the CC domain [153]. Motifs like CC_R_ (resembling RPW8 protein) can also be found [154]. Similarly, the TIR domains are composed of four motifs: TIR1, TIR2, TIR3 and TIR4 [155,156]. The NBS domain itself comprises motifs that mainly interact with nucleotides [19], such as the P-loop (also known as Walker A and Kinase-1a), resistance nucleotide binding site-A (RNBS-A), Walker B (Kinase-3a) and RNBS-C. The hhGRExE [157] motif is a linker region attaching the NBS domain to the CC or TIR domains [152]. There are two motifs on ARC1, namely GLPL [109,155] (also called GxP [157]) and Motif VII [158]; both partake in nucleotide binding [159]. Motifs identified in ARC2 are Motif VIII, RNBS-D, Motif X and MHD [109,155,158]. RNBS-D motif is not consistently present between the TIR and CC domains. It likely co-evolved with their N terminal domain to allow interaction with ARC2 [160]. Figure 1 illustrates the various motifs and their structural organization in some of the most common R proteins.

NBS-LRR proteins are part of the STAND super family with ATPase activity [157]. In the resting or auto-inhibited state, NB-ARC interacts with both its N-terminal LRR and C-terminal CC/TIR domains to maintain a closed conformation [161]. In order to become activated, the LRR domain must be detached from the NBS domain. After detachment, the NB-ARC domain will switch its ADP nucleotides to an ATP to enable rotations within the NB-ARC domain leading to an open conformation [161] allowing the NBS or the N-terminal regions to be exposed for subsequent downstream signaling [161].

**Figure 2:**
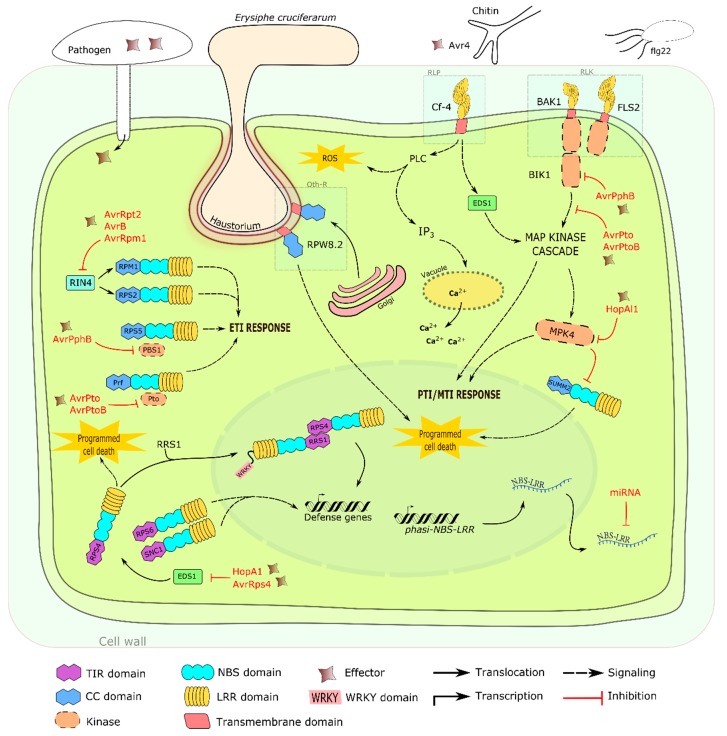
Intracellular signaling mechanisms of RGAs in plant defense. RIN4, PBS1, Pto and EDS1 are targeted and modified by numerous effectors and, as a result, their corresponding TNL or CNL will detect the modification to initiate ETI responses [162,163,164]. TIR-TIR interactions occur between RPS4 and RRS1 to further activate defense genes [169]. Flg22, a bacterial PAMP, activates FLS2 and BAK1 RLKs to initiate the MAP kinase cascade that triggers PTI/MTI responses [170]. MAP kinase cascade signaling can be interrupted by pathogenic effectors. When MPK4 is compromised, SUMM2 will not be inactivated and will initiate PCD [165]. Effector Avr4 is recognized by Cf-4 RLP to initiate MAP kinase cascade and ROS production while simultaneously increasing calcium levels in the cytosol [171]. Upon *Erysiphe cruciferarum* infection, RPW8.2 can translocate from the Golgi to the extrahaustorial membrane where the fungal haustorium has penetrated to activate the downstream signaling of PCD [172,173]. Under normal conditions, NBS-LRR transcripts derived from the *PHAS* locus are regulated through transcript degradation by miRNAs [174]. Such miRNAs include, among others, miR1510, miR1507, miR2109, miR482/2118, miR5668, miR5376, miR172 and miR5041 [174,175,176]. Single arrows may indicate multi-step processes.

As previously mentioned, TNL and CNL proteins recognize pathogen effectors that are secreted into the cell allowing plants to trigger the ETI response. Mechanisms associated with several characterized R proteins and their related ETI responses are summarized in Figure 2. Host protein RIN4 (RPM1-interacting protein 4), guarded by the NBS-LRR encoded proteins RPM1 and RPS2, is targeted by effectors such as AvrRpm1, AvrB and AvrRpt2 [162]. Similarly, enhanced disease susceptibility 1 (EDS1) is also a common target due to its ability to interact with different NBS-LRR proteins such as resistance to pseudomonas syringae 4 (RPS4), resistance to pseudomonas syringae 6 (RPS6) and suppressor of npr1-1, constitutive 1 (SNC1) during their subsequent ETI downstream response [163,164]. Aside from targeting immune regulatory components, effectors can also target PTI/MTI signaling cascades (Figure 2). MAP kinase cascade, specifically MPK4, is capable of suppressing NBS-LRR protein SUMM2 in absence of effector HopAl1; however, when MPK4’s activity is compromised by HopAl1 effector, SUMM2 is activated and initiates hypersensitive programmed cell death (PCD) [165]. ETI and PTI/MTI responses usually result in the production of calcium and phytohormones, oxidative reactive oxygen species (ROS) burst, activation of MAPK cascade (s) [166] and transcription of defense genes to facilitate the hypersensitive response (HR) in order to limit pathogen expansion [167]. Overlaps between the ETI and PTI/MTI defense pathways exist and are important for immune regulation [168]. However, ETI responses have a more heightened downstream effect than PTI/MTI and may also induce PTI/MTI activation in the presence of effectors [168]. This is crucial because effector presence is a true indication of bacterial inhabitancy whereas PAMP/MAMP recognition in PTI/MTI must discriminate between harmful pathogens and beneficial microorganisms [168].

### 2.2. Receptor Like Kinase (RLK) and Receptor Like Protein (RLP) Families

RLK and RLP are main components of the first line of plant immune response triggered by microbial elicitors PAMPs or MAMPs, where the interactions between receptor and elicitor usually take place in the extracellular space. The two proteins are structurally similar with (1) a signal peptide (SP) at the beginning of N-terminus; (2) extracellular domains for perception of the microbial pattern through the leucine-rich repeats and (3) a transmembrane helix domain that can anchor RLP and RLK in the plasma membrane. RLPs differ from RLKs by the lack of an intracellular kinase domain; thus RLPs are unable to independently transduce the perceived signal into a downstream cascade. Both RLPs and RLKs are considered PRRs that recognize elicitors such as lipids, proteins, nucleic acids, and carbohydrates [177].

#### 2.2.1. RLK Family

On the cell surface, plant receptors sense invasion of pathogens and transduce this information through activated signaling pathways to trigger innate immune responses. RLKs are involved in plant development and defense [178]. In plants, serine/threonine protein kinases (STKs) can phosphorylate serine and threonine residues [55]; receptor histidine kinases (RHKs) can phosphorylate histidine residues, e.g., ethylene (ETR1) [179] and cytokinin receptors (CRE1) [180]. TGF β family members represent the only known STK members present in animals [181].

The most well-known RLKs are flagellin sensitive 2 (FLS2) and BAK1 that initiate the MAP kinase cascade upon flg22 recognition [162]. *Xa21* in rice encodes an RLK involved in resistance to a bacterial disease caused by *Xanthomonas oryzae* (*Xoo*) [182]. Several Xa21 binding proteins have been characterized; however, early events governing Xa21 signaling have not been fully elucidated. *XIK1* is an RLK gene whose expression is induced rapidly upon infection with *Xoo*. The reduced expression of *XIK1* compromised disease resistance mediated by *Xa21* [183]. Xa21 binds to a WRKY transcription factor OsWRKY62 [184]. In transgenic plants, the *OsWRKY62.1* variant of *OsWRKY62* is overexpressed in basal defense and in *Xa21*-mediated resistance to *Xoo*. Therefore, *OsWRKY62* function was implied to be a negative regulator of innate immunity in rice, which served as a critical mediator of both basal and race-specific defense responses [184].

#### 2.2.2. RLP Family

Following RLKs, RLPs are the second most abundant RGAs. RLPs have a gene structure similar to RLKs but without a kinase domain (Figure 1). Of note, not all RLPs are involved in disease resistance, some play a role in plant development.

*Cf-9*, the first RLP gene identified, provides resistance against leaf mold induced by the fungus *C. fulvum* [51]. Several *Cf* genes belonging to the RLP family have been isolated from tomato [48,49,50]. Further studies revealed that Cf-9 functions in the cytoplasm by interacting with STK kinase ACIK1 via the CITRX ligand [185,186]. Cf-4, a similar type of RLP, is induced by Avr4 which is an effector that protects chitinous fungi in tomato [187]. This trigger initiates downstream PTI responses via a MAP kinase cascade, ROS accumulation and by releasing calcium ions from the vacuole (Figure 2) [171]. Another type of RLP gene, *CEBiP* isolated in rice, has no typical N-terminal LRR receptor for the perception of elicitor; however, two LysM motifs were observed to perceive chitin oligosaccharide patterns present in fungal cell walls [78], reminiscent of similar discoveries in legume [188,189]. The LysM motif was also noted in the extracellular domains of two legume RLK genes, *NFR1* and *NFR5*, and was suggested to recognize lipochitin-oligosaccharide molecules acting as a nodulation signal triggering plant organogenic processes [188,189]. Thus, the LysM motifs containing PRRs were suggested to function in perception of chitin signals generated by fungi or bacteria [78]. Additional cloned RLP genes involved in plant-pathogen resistance are listed in Table 1.

With respect to RLPs involved in plant development, two typical RLPs, CLAVATA 2 (CLV2) and too many mouths (TMM), were reported to be involved in maintaining meristematic stem cell population balance and regulating epidermis stomatal distribution in *Arabidopsis*, respectively [190,191]. Several studies indicated that CLV2 needed to form a heterodimer with the RLK protein CLV1 via the extracellular ligand CLV3 [192,193,194]. Meanwhile, CLV2 was also required for the stabilization and accumulation of kinase CLV1 [190]. TMM was recently found to interact with both ligands, EPIDERMAL PATTERNING FACTORS (EPFs) and RLK ERECTA, to negatively regulate the development of stomata [195]. These RLPs seemed to be irrelevant to the identification of disease resistance related RLPs. However, it was speculated that these development related genes were much more conserved than those of disease related *R*-genes [109,196]. Based on this hypothesis, Fritz-Laylin *et al.* [110] identified 73 rice disease resistance related RLP genes from a total of 90 RLPs by comparing them to their orthologs in *Arabidopsis*. Therefore, a better understanding of the multiple functions of RLPs and of the divergence as well as conservation between the two types of RLPs will facilitate the identification of the most interesting RLPs of this family.

### 2.3. Oth-R-Genes

The defense system is initiated when extracellular receptors transmit signals to their intracellular partners. To observe and receive these signals, plant cells have complex systems of TM receptors that facilitate communication between the intra- and extracellular environments. However, several reported TM receptors do not contain complete domains as observed in NBS-containing proteins, RLPs or RLKs. Thus, the term oth-R, initially proposed by Walter *et al.*, is used to classify these peculiar RGAs [197]. For example, RPW8.1 and RPW8.2 proteins from *Arabidopsis* contain a putative N-terminal TM domain and a CC domain but lack common NBS, STK or LRR domains [102,198,199]. In response to powdery mildew infection, RPW8.2 is upregulated and trafficked to the target site, *i.e.*, the extrahaustorial membrane, via the *trans*-golgi network [173]. In contrast, RPW8.1 is involved in an assortment of pathogen-mediated responses and, as a result, may induce a broader resistance spectrum in plants [200]. Defense gene *Mlo* also belongs to the TM class that has an intra- and an extracellular loop [39,201]. *LM1*, conferring resistance to stem canker (*Leptosphaeria maculans*) in *Brassica nigra*, is predicted to have two TM motifs [202]. Tomato *Asc1* is an *R*-gene that provides resistance to *A. alternata* [203] and, it encodes a protein with multiple TM domains and a highly conserved motif designated as the Lag1p motif. Asc1 confers insensitivity to the AAL-toxin that inhibits the enzyme sphinganine *N*-acyltransferase involved in sphingolipid metabolism, leading to PCD [204]. *Xa25* is a bacterial blight disease resistance gene in rice that encodes a protein of the MtN3/saliva family, which contains a region of two TM helices [80]. Another type of *Xa10* gene has been characterized in rice as a transcription activator-like (TAL) effector-dependent *R*-gene for resistance to bacterial blight disease [79]. *Rar1* in barley, and *Rtm1*, *Rwm1*, *EDS1*, *NPR1*, *NDR1* in *Arabidopsis* also do not display typical LRR domains involved in pathogen recognition (Table 1).

*Pti1* encodes an STK and acts downstream of *Pto*. It confers resistance to *P. syringae* in tomato, which expresses AvrPto and targets several locations in the cell (Figure 2) [55]. The immune cascade can be initiated through interaction with *Pto* and *Prf* [205,206]. *PBS1* can also be classified as an oth-R gene that has an STK domain and is devoid of any other known functions ([98]; Figure 2). *Fen* is also a member of the STK family that mediates a hypersensitive-like response in tomato plants treated with the organophosphorous pesticide fenthion [53]. *Stpk-V*, cloned from the powdery mildew resistance gene *Pm21* locus [32], is yet another example of STK encoding genes of the Triticeae. Confocal imaging revealed the lack of bias of this protein for its subcellular localization because it was observed in membranes, cytoplasm and nuclei alike [32].

## 3. Other Defense Related Mechanisms

Aside from the typical NBS-LRR and PPR proteins involved in plant defense, recent discoveries led to the description of other modes of defense. Nomura *et al.* [207] demonstrated that chloroplasts are an important component in activation and signaling of immunity. Upon exposure to flg22, chloroplast calcium-sensing receptor (CAS) dependent immune resistance and PCD are initiated [207]. Likewise, mitochondria play a similar role [208], highlighting the importance of these organelles in the defense pathways activated in response to pathogen attacks.

Chloroplast and mitochondrial transmigrated proteins have been described [207,208]. They belong to the PPR protein class that constitutes one of the largest protein families in plants. Intriguingly, PPR proteins have undergone an evolutionary process similar with the above-mentioned LRR associated proteins [209]. Characteristics such as gene clustering and duplications within clusters have been observed [209]. PPR proteins are nucleus-encoded and will translocate to the chloroplast and mitochondria to perform post transcript processing such as RNA editing, splicing and translation modification [210].

Known PPR genes, such as *RPF2 and RPF3* in *Arabidopsis*, *Rf1* in rice and *Rf2* in maize have been identified ([211,212,213,214]; Table 3). In addition, an increase in *PPR1* (At1g06580) and *PPR2* (At1g62720) transcripts was observed in response to pathogen attack [215]. Although their roles were not clearly illustrated, these proteins were speculated to be involved in mitochondrial ROS metabolism [215]. Likewise, Garcia-Andrade *et al.* [216] identified a negative regulation of PPR protein chlororespiratory reduction21 (CRR21), PPRα (at4g21190) and overexpressor of cationic peroxidase3 (OCP3) in response to chitosan, a fungal PAMP/MAMP. These proteins correspond to chloroplast *NADH dehydrogenase-like complex* (*NDH*) transcript editing. NDH-mediated immune response remains inconclusive; however, ROS production and callose deposition were speculated to contribute to pathogen-mediated resistance [216].

**Table 3:** Other cloned genes relevant to plant defense.

Species	*R-Gene*	Accession ID	Domain ^a^	Chr	Disease	Avr	*Pathogen* ^b^	Reference
*H. vulgare* (barley)	*Rar1*	AAF18432	CHORD	2	Powdery mildew		*B. graminis*	[217]
*S. lycopersicum* (tomato)	*Asc*	AAF67518	TLC	3	Black mold rot; Black shoulder		*A. alternate*	[218]
*O. sativa* (rice)	*Rf1*	BAC77666	PPR	10				[214]
	*LYP4/6*		TM				*X. oryzae*; *M. oryzae*	[219]
*Z. mays* (maize)	*Rf2*	AAC49371	PPR	9				[213]
*A. thaliana* (*Arabidopsis*)	*RPF2*	NP_176454	PPR	1				[211]
	*RPF3*	NP_176481	PPR	1				[212]
	*Rtm1*	AT1G05760	Jacalin like	1	Tobacco etch		TEV	[220]
	*Rwm1*	AEE33357	PGK	1	Mosaic type		WMV	[221]
	*EDS1*	AAD20950	Lipase-like	3		*AvrRps4*	*P. syringae*	[222]
	*NPR1*	AAC49611	Ankyrin	1			*P. syringae*	[223]

^a^: CHORD, cysteine and histidine-rich domain; PGK, phosphoglycerate kinase; TLC, tram-lag1-cln8; TM, transmembrane; ^b^: *A. alternata*, *Alternaria alternata*; TEV, Tobacco etch virus; WMV, watermelon mosaic virus.

As mentioned above, chloroplast and mitochondria, involved in the production of ROS, activate defense and constitutively initiate PCD [224]. Various levels of ROS are produced at different concentrations in response to diverse pathogens [224]. It is intriguing to note that aside from these organelles and oxidase enzymes, about half of the ROS level is generated exclusively from peroxidases upon PAMP/MAMP recognition [225]. Apoplastic peroxidases, PRX33 and PRX34, have been well studied in response to flg22 and EF-Tu [225,226,227]. Aside from generating hydrogen peroxide (H_2_O_2_), they play roles in callose deposition and MAMP/PAMP-mediated transcription of defense genes as well [225,226]. Therefore, peroxidases are important for plant immunity.

In addition, small RNAs have been found to play a major role in defense, especially in regulating immune components in the cell [174]; however, further studies are needed as their mechanisms remain poorly characterized.

## 4. Bioinformatics Approaches for RGA Identification and Characterization

To date, genome sequences of more than 50 plant species have been sequenced and assembled to various degrees [19,20,228]; the released sequences were deposited in public databases such as Phytozome [19,20] and EnsemblPlants FTP servers [229]. Advances in next generation sequencing technologies have made whole genome sequencing one of the most important approaches in modern biological research. Current challenges include the provision of functional annotations for the large number of macromolecules. However, experimental investigations to assign protein functions are costly and time consuming. Alternatively, computational approaches to functional prediction are very attractive to solve this complex task [230]. Mining and characterizing genome-wide plant RGAs using computational approaches are rendered possible due to their significant structural features and conserved domains. Several bioinformatics methods have been applied to identifying RGAs and predicting their functions, including sequence alignment, BLAST search, phylogenetic analysis, and domain and motif analysis [231] using several applications such as Hidden Markov Model (HMM) [232], SMART (http://smart.embl-heidelberg.de/) [233], Prosite (http://prosite.expasy.org/), pfam (http://pfam.xfam.org/), and InterProScan5 (http://www.ebi.ac.uk/Tools/pfa/iprscan5/) which are summarized (Table 2 and Table 4).

**Figure 3:**
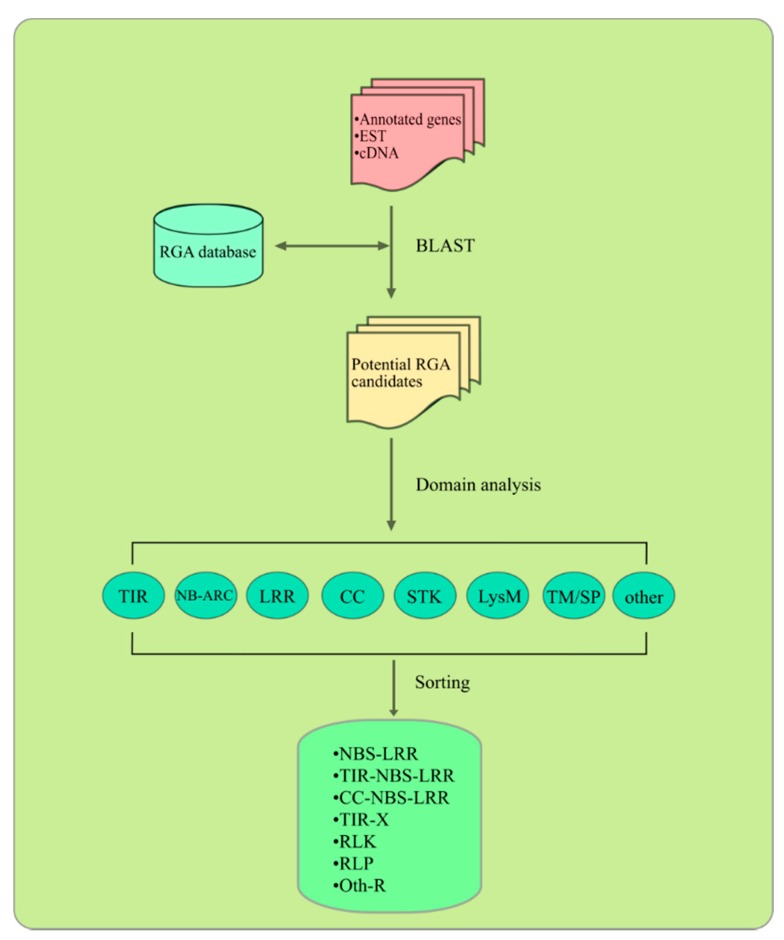
A common procedure for identification and characterization of plant RGAs.

Based on previously used approaches, the identification and characterization of RGAs usually follow a common procedure of four steps (Figure 3). First, a plant RGA database including all known plant RGA gene and protein sequences is generated. GenBank [234] and PRGdb [235] are two important sources of well curated RGA sequences. Second, BLAST searches against the RGA database are performed using a loose E-value cut-off (from 1e-5 to 1 depending on the genome size) to identify RGA candidates. Third, using the RGA candidates as input, a variety of software tools (Table 4) are employed to detect various conserved domains and motifs and produce alignments. Some programs like pfam_scan.pl (developed by Sanger) and InterproScan can be run in a parallel mode. In the last step, a dedicated sorting script is needed to group the RGA candidates into classes as per their domain and motif structures or a combination thereof. For example, to be classified as a gene encoding a TNL protein, an RGA must have a 5′ TIR and an NB-ARC followed by an LRR domain.

To date, no standardized bioinformatics tools and consistent annotation criteria were employed in individual studies. Also, individual software tools may have their own advantages and limitations in identifying different types of RGA domains. Thus, the results from different studies are not necessarily comparable. A comprehensive pipeline package to seamlessly integrate these individual tools is expected to save biologists’ time by facilitating processing, standardizing data organization and providing visualization features. The use of consistent criteria to identify the complete RGA complements would permit their comparative analyses across species.

**Table 4:** Common software used for RGA domain and motif identification.

Software	Latest Version	Input Type ^a^	Required Database	Description	Parallel Support ^b^	URL ^c^	Reference
HMMER	3.1b2	D/P	HMM model	Protein or DNA sequence homolog search toolkits using profile hidden Markov models and featured by remote homolog identification. The latest version is as fast as BLAST thanks to the underlying mathematical models.	HT/MPI	hmmer.janelia.org	[236]
MEME	4.10	D/P		Discover novel and ungapped motifs from nucleotide or protein sequences without well trained dataset samples.	MPI	meme-suite.org	[237]
mCUDA-MEME	3.0.15	D/P		An ultrafast scalable motif discovery program running on graphics processing unit (GPU). The algorithm is based on MEME using a hybrid combination of CUDA, MPI and OpenMPI parallel programming models.	CUDA/MPI	bit.ly/18X8LmA	[238]
BLAST+	2.30	D/P	BLAST databases, like nr or nt database	Classical similarity search toolkits for bioinformatics data mining. The latest version significantly improves the speed on CPU and efficiency on RAM for long queries.	HT	blast.ncbi.nlm.nih.gov	[239]
pfam_scan.pl	1.0	P	Pfam-A HMM model	A Perl script for PFAM database search, which invokes “hmmscan” in the HMMER toolkit package to search known HMM models.		bit.ly/1M41KRu	
InterproScan	5.9	P	PFAM, SMART, PANTHER, PROSITE, Superfamily, *etc*.	A tool that combines different protein signature recognition methods native to the InterPro member databases into one resource with lookup of corresponding InterPro and GO annotations.	HT	www.ebi.ac.uk/interpro	[240]
Phobius	1.01	P	HMM model	A HMM based tool for transmembrane (TM) topology and signal peptides (SP) prediction from proteins. A pre-training HMM model has been embedded in the tool.		phobius.sbc.su.se	[241]
TMHMM	2.0	P	HMM model	A HMM based tool with similar functions to Phobius.		www.cbs.dtu.dk/services	[242]
nCOILS	2.2	P	Scoring matrix	A program to detect CC domains by comparing and scoring protein sequences with a known coiled-coils database with the MTK or MTIDK calculation matrix, which reports a probability that the sequence adopts a coiled-coil conformation.		embnet.vital-it.ch	[243]

^a^: D, nucleotide; P, amino acid; ^b^: HT, hyper-thread; MPI, message passing interface; CUDA, a computing platform implemented by nVIDIA on GPUs; ^c^: abbreviated bitly URL links were used to replace real URL, case sensitive.

## 5. Genome-Wide Identification and Characterization of RGAs

Whole genome sequencing of plants has enabled genome-wide identification, mapping and characterization of RGAs. NBS-LRR containing RGAs have been evaluated in numerous plants such as apple, *Arabidopsis*, barley, black cottonwood, grape, maize, *Medicago*, rice and sorghum (Table 2). Hundreds of NBS-LRR encoding genes have been identified from these plant genomes. NBS-LRR genes are a highly duplicated, evolutionarily diverse and clustered gene family [244] and, they represent the major class of *R*-genes contributing to disease resistance in plants [245]. Angiosperms possess NBS-LRR encoding genes but TNL encoded genes are absent from grass genomes [196,246] and other monocots [114]. It has been hypothesized that this absence is likely due to either a loss or the lack of amplification of TNL encoding genes in the monocot lineage [156,247,248]. The loss of TNL-encoding genes in monocots could be due to increased dependence on CNL proteins [156]. In rice, CNL proteins are encoded by many more genes than the total number of CNL and TNL genes in *Arabidopsis* [156]. More NBS-LRR and CNL encoding genes have been identified in most monocots than in dicots (Table 2). CNL and TNL proteins may utilize different downstream signaling components in disease resistance responses [249]. This genomic shift to CNL genes in monocots could have resulted from mutations in genes encoding downstream components of TNL-specific pathways, resulting in a loss of function and conservative selection for TNL genes. Therefore, TNL-encoding genes may have been lost or simply never amplified in monocot genomes due to a lack of selective advantage [156]. In addition, some dicots like *Arabidopsis* contain more TNL than CNL [109,118]. The abundance of TNL likely results from its older origin than CNL [250]. Besides TNL and CNL, variants of NBS encoding genes such as CN, NL, TN and N vary largely in number (Table 2). Other NBS-LRR like domains such as CTNL and CTN in apple [17], TN-TNL, TTNL, XTNX and SA-CA (signalling commentator with CC domain, e.g., RPW8) in *Arabidopsis* [251], and, CNLX, CNX, CNXL, CXN, NX and NLX in sorghum [138], were reported. Numbers of TIR-X RGAs were also reported such as 126 in cabbages, 46 in *Arabidopsis*, 67 in cottonwood and 92 in *Medicago* [111].

More than 600 members of RLKs were identified in *Arabidopsis* [112] and around 1200 members in rice [132]. They have also been reported in maize, wheat, tomato and cottonwood (Table 2). RLPs with TM domain have also been reported in *Arabidopsis* and tomato (Table 2). Some cloned genes, such as *Hs1pro-1* in sugar beet, *Cf2*, *Cf4-9*, *Ve1* and *Hcr9-4E* in tomato, belong to this class (Table 1).

Some RGAs have been identified as pseudogenes. A total of 49 *R*-pseudogenes in *Medicago* [16], 179 in potato [124], 347 in the rice variety “Nipponbare” and 345 in variety “93-11” [252], 10 in *Arabidopsis* [109], 161 in cottonwood [115] and 62 in lotus [128] have been identified. In tomato, only 10% of the cloned RGAs were classified into pseudogenes [253], but almost half of the identified RGAs were pseudogenes in western white pine (*Pinus monticola*) [254]. Pseudogene paralogs of several R-genes such as *Xa21*, *Cf9*, *Pto* and *Dm3*, were also identified [254]. Most identified pseudogenes have strong identity with another NBS protein but their sequences are shortened by premature stop codons or frameshift mutations. Sequence comparisons have suggested that RGA pseudogenes originated from point mutations, like insertion or deletion of nucleotides [254]. Usually, pseudogenes are considered non-functional genes; however, some pseudogenes can be transcribed into mRNA [254,255]. Evidence of expression also exists in species such as rice [256], pine [254] and *Medicago* [16]. In these species, some pseudogenes have 90%–100% identity to ESTs and their functions are ascribed as partial NBS-LRR proteins. Pseudogenes are believed to power *R* gene evolution by driving illegitimate recombination and permitting gene conversion with alleles or paralogs of functional *R*-genes [257].

As noted earlier, there is surprising similarity in the *PPR* genes of *Arabidopsis* and rice supporting their existence prior to the divergence of monocots and dicots [130]. However, massive differences between the numbers of *PPR* genes in higher plants and non-plant organisms are indicative of the expansion of this gene family during the evolution of the plant kingdom [130].

**Figure 4:**
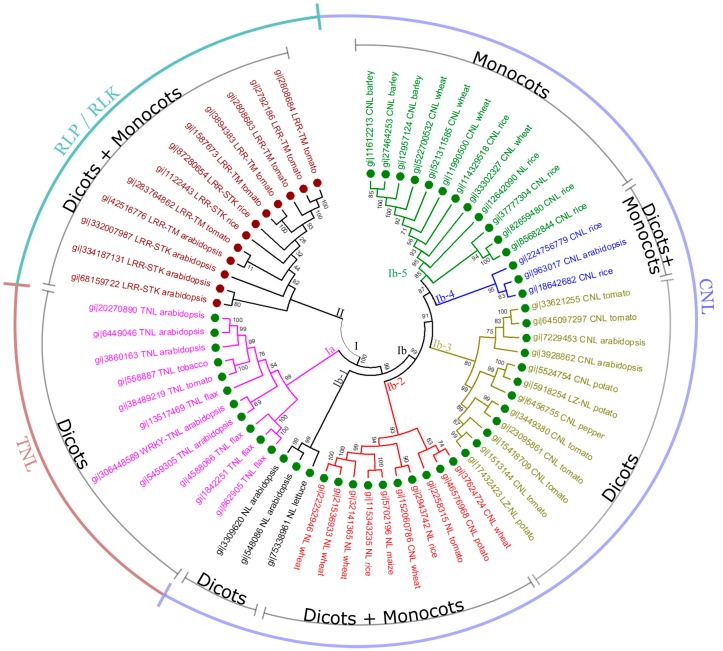
Phylogenetic analysis of RGAs in plants. The protein sequences of 63 RGAs or cloned *R*-genes from eleven plant species were selected for this analysis using MEGA 6 [258]. The protein sequences were aligned using the Muscle algorithm, and then clustered using the neighbor-joining algorithm with the p-distance model, pairwise deletion for gaps or missing data treatment, and 500 bootstrap replicates. The phylogenetic tree consists of two large clades (Clades I and II), representing the NBS-LRR class and the RLK/RLP class of proteins, respectively. Clade I may be divided into two sub-groups (Clades Ia and Ib), containing TNL and CNL proteins, respectively, while Clade Ib may be further split into several diverged CNL clusters. The bootstrap values are labelled on branches.

Some RGAs represent a highly divergent defense system in plants, containing a large and diverse family of genes with conserved motifs and domains [254]. To understand the phylogenetic relationship of RGAs, we selected 63 well-characterized RGAs or cloned *R*-genes (Table 1) in seven dicot (*Arabidopsis*, black pepper, flax, lettuce, potato, tobacco and tomato) and four monocot species (barley, maize, rice and wheat) from the NCBI database. These selected RGAs represent the major classes of RGAs: NBS-LRR (CNL, TNL or NL), RLK (LRR-STK) and RLP (LRR-TM). A neighbor-joining tree with 63 RGAs was constructed using MEGA 6 [258] (Figure 4). RGAs were classified into two groups: Clade I for NBS-LRR encoding genes and Clade II for RLK and RLP encoding genes. As RLP and RLK proteins have common domains but differ in presence or absence of a kinase (Figure 1B), LRR-STK and LRR-TM encoding genes clustered into one large group (Clade II) with sub-clusters. CNL and TNL are major subclasses of NBS-LRR encoding genes; thus they grouped into distinct sub-clusters in Clade I (Clades Ia for TNL and Ib for CNL encoding genes, respectively) (Figure 4). We observed that TNL encoding genes were conserved among different dicot species (Clade Ia). In contrast, CNL encoding genes are much more diverse. Some of them are conserved within angiosperms (Clades Ib-2 and Ib-4), within dicots (Clades Ib-1 and Ib-3) or within monocots (Clade Ib-5), while others have diverged between dicot and monocot species (Clade Ib) or within dicots (between Clades Ib-3 and Ib-5). For example, Clade Ib-3 contains RGAs from dicot species only while Clade Ib-5, from monocot species only. In addition, NL and LZ-NL encoding genes may be more closely related to CNL than to TNL because almost all NL and LZ-NL encoding genes clustered with the CNL clades (Clades Ib-1, Ib-2, Ib-3, Ib-4 and Ib-5), suggesting that the variants of non-TNL, such as NL and LZ-NL, may have evolved from CNL rather than TNL.

## 6. Genome Organization of RGAs

Many studies revealed that RGAs are irregularly distributed in plant genomes and that many reside in clusters. The clustered distribution of RGAs provides a reservoir of genetic variation to drive the evolution of new *R*-gene specificities [257,259,260].

Most RGAs are found in clusters. For example, 38.2% of the mapped NBS genes were located into eight clusters in the lotus genome [128]. Similarly, 50% and 51% of NBS genes were clustered in the rice [261] and *Brachypodium* genome [141], respectively. Higher rates have been found in other species such as potato where 73% of the mapped NBS-LRR genes grouped into 63 clusters [262], while approximately 80% were clustered in *Medicago* [16], 74.3% in the rice cultivar “Nipponbare” and 71.1% and 63.8% in *Arabidopsis* and lyrata, respectively [114]. Many super**-**clusters were identified for NBS-LRR genes, including a super**-**cluster with 11 NBS genes on chromosome 4 of *Brachypodium* [141] and one with 82 NBS-encoded genes in *Medicago* [16]. In addition, some *R*-genes appear in complex cluster structures [257] forming a diverse multigene family such as *Cf4/9* in tomato [49], *L* in flax [14], *Mla* in barley [263], *Pi2/9* in rice [260], *Dm3/13* in lettuce [60] and *I2* in tomato [42]. The structures of three haplotypes (linked genes) of the *Pto* cluster in tomato have been defined [264] while a single haplotype of the *Xa21* cluster in rice was partially characterized [265]. Several restorer genes, like the *Rf* gene from various plant species, showed homology to a cluster of *PPR* genes [266]. Genes within a single cluster may determine resistance to different pathogens [257].

RGAs are often irregularly distributed on chromosomes. Gebhardt *et al.* [267] and Lozano *et al.* [124] published genetic maps of potato with resistance traits. Their analyses indicated that large numbers of NBS-LRR genes were found on chromosomes 4 and 11 (approximately 15% of the mapped genes), while only 1% was on chromosome 3. In *Medicago*, chromosomes 6 and 3 encoded approximately 34% and 40% of all TNLs, respectively [16]. In apple, 56% of the 868 identified RGAs were distributed on six chromosomes (Chr 2, 7, 8, 10, 11 and 15) including 25% on Chr 2 while 99% of the non-TNL class was on Chr 11 [268]. Pseudogenes are also distributed and clustered at specific chromosomal locations in the same way as functional NBS-LRR genes [16,124]. Pseudogenes evolve much faster than functional genes and represent a reservoir for evolution of new specificities [269].

In conclusion, genetic and molecular data have shown that disease resistance genes are frequently clustered in plant genomes. Several cloned *R*-genes have common domains which will help to identify disease resistance loci from clusters of paralogs [257]. *R* loci may harbor single genes with multiple alleles, for instance, the *L* locus in flax with 13 alleles or *RPM1* in *Arabidopsis* with two [257]. However, some resistance loci and clusters of resistance genes are inherently unstable, e.g., *Rp1* in maize was described as a fast evolving complex [270].

## 7. Applications of RGAs

### 7.1. RGAs Are R-Gene Candidates for Disease Resistance

Map-based cloning remains the major strategy for isolating resistance genes [23,28,29,44,54,65], a strategy that requires high-density genetic maps. Genome-wide RGA identification will facilitate the development of molecular markers towards *R-*gene mapping and cloning. To date, all cloned disease resistance genes in plants belong to several major classes of RGAs (Table 1). Genome-wide RGAs can be interrogated as *R*-gene candidates. In sorted and assembled genome sequences, the physical location of the identified RGAs can be easily retrieved. Single nucleotide polymorphism (SNP) markers can be designed from RGAs around a target disease gene to construct an RGA genetic map for the specific target region. These mapped genome-wide RGAs and SNP markers in their vicinity are valuable tools to develop high density *R-*gene genetic maps, target *R*-genes, co-localize QTL, design diagnostic markers of *R*-genes for fine mapping and cloning of *R*-genes and for resistance breeding.

### 7.2. Genome-Wide Association Study (GWAS) with Mapped RGAs Helps Co-Localization of QTL to Resistance Genes

Linkage mapping has been a key tool to identify resistance genes in the past 30 years. However, linkage mapping is sometimes limited by population size or low frequency of recombinant events near the target genes of interest resulting in increased expense and gene cloning difficulties. Under this scenario, association mapping, an application of linkage disequilibrium (LD), was developed to address these issues [271]. Today, modern genotyping techniques such as genotyping by sequencing (GBS) and chip-based genotyping facilitate GWAS for qualitative gene and QTL mapping. The primary advantage of GWAS over linkage mapping is the unprecedented high resolution across the whole genome. Consequently, population size for fine mapping may not need to be as large as for linkage mapping. With the aid of the identified and mapped genome-wide RGAs, more genes or QTL associated with disease resistance are able to be fine-mapped and co-localized.

Along with the development of comprehensive plant haplotype mapping projects in different crops such as *Arabidopsis* (http://1001genomes.org/) [272], flax (http://tufgen.ca), rice (http://ncgr.ac.cn/ricehap3/) [273], wheat (http://wheatgenome.org) [274] and maize (http://panzea.org) [275,276], a large number of crop varieties, ecotypes or breeding lines have been resequenced. The sequencing information of most projects has been released to the research community. These projects provide biologists with an invaluable blueprint to exploit SNP and indel markers, comparable to the prestigious human 1000 Hapmap project [277] (http://1000genomes.org). These plant haplotype maps permit the establishment of relationships between RGAs and plant disease resistance, providing precise sequence information to design diagnostic markers for breeding and to identify *R-*genes.

### 7.3. RGA Mapping in Plants with Limited Genome Information

In addition to the traditional marker types to construct genetic linkage maps such as random amplified polymorphic DNA (RAPD), restriction fragment length polymorphism (RFLP), cleaved amplified polymorphic sequence (CAPS), amplified fragment length polymorphism (AFLP), simple sequence repeat (SSR) and SNP markers, specific methods have emerged from the identification of RGAs. NBS profiling is a useful and practical linkage map construction method based on genetic markers that has been implemented in potato [278]. NBS profiling was first used to map RGAs in cauliflower where a genetic linkage map was constructed based on the combined use of AFLP and NBS profiling [279]. Linden *et al.*, have described an advanced NBS profiling approach based on conserved NBS amplification in several crop plants such as potato, tomato, barley and lettuce [278].

Degenerated primers have been designed to clone NBS genes according to their conserved domain structure using PCR. With a properly constructed mapping population such as a doubled haploid (DH) or a recombinant inbred line (RIL) population, an NBS linkage map can initially sort out these markers by linkage groups or chromosomes. This method is used when the whole genome has yet to be sequenced and assembled because it directly associates the markers with the target gene class. Furthermore, with proper modification of the degenerated primers design, NBS profiling can also be extended to other RGAs as long as the gene family of interest contains substantial members across all chromosomes. Therefore, although many species of interest have already been sequenced and large numbers of SNPs have been identified in these species, NBS profiling remains a powerful tool for the development of markers linked to resistance loci in species with limited genome information. A similar profiling method for other *R*-gene classes, like peroxidase profiling, developed in barley, revealed the resistance of *R-*genes for rusts and mildew [280]. RLK and LRR profiling strategies in potato were also developed [281]. Meanwhile, other non-RGA gene families can also benefit from this idea, like MYB profiling in pot azalea [282].

## 8. Conclusions

Plant RGAs are a large group of potential *R*-genes that have conserved domains and structural features which have specific roles in host-pathogen interactions. Bioinformatics software tools and comprehensive pipelines will help in their identification and characterization. Numerous RGAs have been identified from several sequenced plant genomes. These identified genome-wide RGAs with applications in genomics and bioinformatics such as linkage mapping, GWAS, clustering and protein signature profiling will assist traditional methods to enhance marker development, QTL mapping, cloning of plant resistance genes and resistance breeding.
